# The Relationship between Allostasis and Mental Health Patterns in a Pre-Deployment French Military Cohort

**DOI:** 10.3390/ejihpe11040090

**Published:** 2021-10-12

**Authors:** Marion Trousselard, Damien Claverie, Dominique Fromage, Christel Becker, Jean-Guillaume Houël, Jean-Jacques Benoliel, Frédéric Canini

**Affiliations:** 1 Département Neurosciences & Sciencs Cognitives, Institut de Recherche Biomédicale des Armées (IRBA), CEDEX, 91223 Brétigny-sur-Orge, France; claveriedamien@hotmail.com (D.C.); dominique.fromage@gmail.com (D.F.); frederic.canini@intradef.gouv.fr (F.C.); 2 Ecole du Val de Grâce, 1 place A. Laveran, 75005 Paris, France; 3 APEMAC EA 4360 UDL, 57000 Metz, France; 4 Faculteé des Sciences Fondamentales et Biomeédicales, Universiteé de Paris, INSERM UMRS 1124, 45 Rue des Saints-Pères, 75006 Paris, France; christel.becker@sorbonne-universite.fr (C.B.); jean-jacques.benoliel@upmc.fr (J.-J.B.); 5 11ème Centre Médical des Armées, 10 rue Roquemaurel, 31300 Toulouse, France; jghouel@gmail.com

**Keywords:** allostatic load, BDNF, glucocorticoid, military, oxidative stress, psychopathology, stress response

## Abstract

(1) Background: While a number of studies among military personnel focus on specific pathologies such as post-traumatic stress disorder (PTSD), anxiety, and depression, they do not address the cumulative impact on mental health of stressors related to the profession. The present study aims to determine the relationship between allostatic load and mental health status in a cohort of fit-for-duty soldiers prior to their deployment to Afghanistan. The aim is to better-define the consequences of stressor adjustment. (2) Methods: A cohort of 290 soldiers was evaluated in a cross-sectional study with respect to psychopathology (PTSD, anxiety, depression), psychological functioning (stress reactivity, psychological suffering), and allostatic profile (urinary cortisol and 8-iso-PGF2α, blood cortisol and BDNF). A hierarchical cluster analysis was used to identify allostatic patterns. (3) Results: Around 10% of the cohort reported high scores for psychopathology, and biological alterations were identified. For the remainder, four allostatic profiles could be identified by their psychological functioning. (4) Conclusions: Both biological and psychological assessments are needed to characterize subthreshold symptomatology among military personnel. The psychological significance of allostatic load should be considered as a way to improve health outcomes.

## 1. Introduction

Not only are military personnel exposed to stressful events in civilian life, they must also cope with high-level stressors when deployed in overseas theatres. Private and professional stressors are repeatedly combined, leading some personnel to experience physiological [1], and psychological impairments [2]. These pathologies can be moderate in terms of frequency or severity [3,4,5,6], or be more disabling [2,7,8,9,10].

Regardless of the medical outcome, which is a function of the individual, the relationship between exposure to stressors and impaired health deserves discussion. The understanding of stress biology has largely evolved over the past decades [11]. The biological framework allows to broaden the well-known three stages of the general adaptation syndrome model by taking into account the biological cumulative impact of stressor exposure on health outcomes [11,12,13]. In this context, the leading mechanism that is thought to underlie the relation is repeated, stress-induced allostasis [11]. This concept, initially called heterostasis [12], refers to the high-cost mode of functioning of an organism under stress, which is very different to the usual, economical state of homeostasis [13,14,15]. Chronic strain and life events increase the allostatic load. The latter reflects the functional and structural cost of stress [14,15], and is an indicator of the essential protective and adaptive effects of the physiological mediators that maintain homeostasis, or their cumulative impact on daily life if they are mismanaged or overused [16,17]. The wide-ranging impacts of allostasis have resulted in its integration into studies of physiological regulation in response to psychosocial and socioeconomic stressors, notably with respect to how adjustments are made to minimize the latter’s impact [18]. Several biological mediators of allostatic load have been identified [19]. Among them, cortisol, oxidative stress, and brain-derived neurotrophic factor (BDNF) are three, key independent factors [19,20].

The physiological response to a challenge is shaped by the concomitant stress response and its biological sources. The stress response can be described in terms of both allostatic load and tissue tropism. It is mainly seen in cortisol levels, as cortisol is the main hormone controlled by the hypothalamic–pituitary–adreno–cortical (HPA) axis, and is a biomarker of both the stress response and allostasis [21,22]. Nocturnal urinary excretion of cortisol reflects the basal tone of the HPA axis [23], and provides information on the quality of HPA inhibitory feedback. Conversely, blood cortisol concentration, measured in a challenging environment, is an indicator of stress reactivity [24].

The accumulation of free radicals is another marker of allostatic load [21,25]. Free radicals can be indirectly detected by a large panel of biomarkers, notably 8-iso-prostaglandin F2α (8-iso-PGF2α) [26], as the latter increases in chronic stress [27] and depression [28]. Neurotrophic factor production also plays a role in allostasis by protecting neurons [20,29]. In particular, BDNF increases with moderate stress [30], but decreases with high-level stress [31]. Therefore, it is possible to define the adaptative physiological response to an environmental challenge based on nocturnal urinary cortisol excretion, morning blood cortisol concentration, 8-iso-PGF2α, and BDNF.

Although allostasis has been applied in a number of biomedical contexts, few studies have attempted to use allostasis mediators to connect biomedical and ecological data. Studies have focused on pathologies such as post-traumatic stress disorder (PTSD), which may occur after exposure to a highly stressful event that induces an intense reaction, and anxiety and depression that may occur after repeated exposure to non-traumatic life events [32,33]. However, these studies do not address the consequences of exposure to a stressor. In practice, the majority of people who are exposed to stressors in their private or professional life only present infra-clinical psychological suffering, and no psychopathology. However, biological scars may still be detected in people who have been exposed to stressors, despite their lack of clinical symptoms. Identifying this specific state may be an important way to protect soldiers from further psychopathologies. Therefore, we focus on biological pathways for stress become more primed and prepared for future stress, in turn leading to one’s resting allostasis geared toward higher maladaptive patterns of reactivity [34]. We hypothesize that the four markers of allostatic load noted above can be used to characterize certain stress-related psychiatric conditions, and identify abnormal patterns in a healthy population.

The main objective of our study is, therefore, to determine the allostatic load in a fit-for-duty cohort of soldiers preparing for deployment. In particular, we consider the groups’ psychometric and psychopathological status (whether they present a psychopathology or not), with two objectives. First, we aim to evaluate the allostatic load of soldiers diagnosed as suffering from PTSD, anxiety, or depression based on nocturnal urinary cortisol, morning blood cortisol, 8-iso-PGF2α excretion, and BDNF concentrations. We hypothesize that among mission-ready soldiers, those with high scores of psychological suffering suffer from a higher allostatic load than those with low scores. Second, we aim to determine the allostatic correlates of the psychological profiles of soldiers who do not suffer from PTSD, anxiety, or depression. Our hypothesis is that there are different biological profiles of allostasis characterizing different psychological profiles.

## 2. Materials and Methods

### 2.1. Population

The study was conducted in a population of 405 soldiers in the French army who were scheduled for a six-month deployment in Afghanistan in the spring of 2011. Inclusion criteria were having volunteered to participate in the study, being aged between 18 and 50, and being medically fit for military deployment. There were no exclusion criteria. Recruitment took place during pre-deployment training. The study was approved by both the French Armies’ Health Service Ethics Committee, and the French Health Authority (under number 2010-A01232-37). In compliance with the Helsinki Convention that controls and regulates experiments on humans, informed consent was obtained from all participants.

### 2.2. Protocol

We adopted a cross-sectional ecologic design. The objectives of our investigation were presented by military health authorities during a briefing that was carried out approximately one month before deployment. Participants were asked to collect their urine between 22:00 and 06:00, and to report the following morning for blood collection and psychological assessment. They were asked to not practice sport, drink coffee, or smoke in the two hours preceding blood collection. Blood was collected between 08:30 and 10:30 to control for circadian variation, and after ten minutes spent relaxing. Participants also completed a set of paper-and-pencil standardized assessments that captured sociodemographic data, and details of psychological and pathological functioning. These assessments took approximately one hour to complete.

### 2.3. Biological Variables

The volume of urine samples was measured, and 2 mL extracts were collected and stored at −80 °C until analysis. Urinary cortisol (U-CORT) concentrations were measured using enzyme-linked immunosorbent assay kits (IBL International GMBH, Hamburg, Germany). Urinary 8-iso-PGF2α (U-PGF) concentrations were measured using enzyme-linked immunosorbent assay kits (Eurobio, DRG, Heidelberg, Germany). Urinary excretion was calculated according to diuresis and creatinine excretion rates.

Blood samples were clotted and centrifuged, while plasma and serum were sampled into 1.5 mL aliquots that were stored at −80 °C until analysis. Plasma cortisol (B-CORT) concentrations were analyzed using enzyme-linked immunosorbent assay kits (IBL international GMBH; Hamburg, Germany). Serum BDNF (B-BDNF) concentrations were determined at a dilution of 1:10 with a commercial BDNF assay (Promega Corporation, Madison, WI, USA) in 96-well plates (Corning Costar^®^ EIA plate, New York, NY, USA). All tests were run in duplicate and according to the manufacturer’s instructions.

### 2.4. Psychological Variables

#### 2.4.1. Sociodemographic Evaluation

Sociodemographic variables included age, gender, marital status, tobacco use, experience (measured as time served), previous overseas deployments (if any), and, if so, the number of deployments.

#### 2.4.2. Psychopathological Evaluation

Although all members of the cohort had been declared healthy following a medical examination, they completed the Hospital Anxiety-Depression Scale (HAD), and the Posttraumatic Stress Disorder Check List (PCL) to evaluate their adaptation to the environment. The HAD consists of two subscales that aim to detect anxiety (HAD-A) and depression (HAD-D) in general, non-psychiatric medical outpatients [35,36]. A cut-off of ≥11 was chosen in order to prioritize specificity (0.92) over sensitivity (0.56) [37]. Internal consistency was acceptable (Cronbach alpha between 0.67 and 0.68 for HAD-D and HAD-A, respectively). The PCL was used to detect PTSD based on DSM-IV-TR criteria [38]. A cut-off of ≥44 was chosen to optimize sensitivity (0.864) and specificity (0.944) [39]. However, this value may overestimate the prevalence of PTSD [40]. Internal consistency was good (Cronbach alpha: 0.94).

#### 2.4.3. Psychological Evaluation

Stress reactivity was assessed using four questionnaires. Perceived stress was evaluated using the validated French version [41,42,43] of the Perceived Stress Scale (PSS) [41]. The PSS is a self-report measure of the degree to which the respondent has perceived stressful situations in his/her life in the past month. Alexithymia was assessed using the validated French version [44] of the Toronto Alexithymia scale (TAS) [45,46], where a score below 44 indicates no alexithymia [47]. Trait anxiety was measured using the validated French version [46] of the Spielberger Trait Anxiety Scale (STAI-T) [48]; scores over 42 are considered to be high. State anxiety was evaluated using the validated French version [49] of the Spielberger State Anxiety Scale (STAI-S) [48]; scores over 35 are considered high. For these assessments, internal consistency was good (Cronbach alpha between 0.78–0.89).

Mental health was evaluated using four measures. The Burnout Measure Short version (BMS) evaluates the level of burnout [50,51]. Developed for use with all occupational groups, it is well-suited to a military population. No cut-off is defined in the French version [51]. The Positive and Negative Affect Scale (PANAS) [52]) is a good index of distress. Scores above 33.3 indicate positive affect (PA), and scores above 17.4 suggest negative affect (NA) [53,54]. Finally, participants completed the validated French version [53] of the 28-item General Health Questionnaire (GHQ28) usually used in healthy populations [55,56]; a score over 22 indicates psychological distress [57]. For these assessments, internal consistency was good (Cronbach alpha between 0.74–0.9).

Questionnaires were excluded from further analysis if more than two items were not completed. If only one item was not completed, its value was considered to be the mean of the other items.

### 2.5. Statistical Analysis

All statistical analyses were performed using Statistica software (Stastsoft France, Maison Alfort, France, version 7.1). Clustering was carried out using SPSS software (SPSS INC, Chicago, IL, USA, version 24.0).

The relation between the four biological variables (U-CORT, U-PGF, B-BDNF, and B-CORT) was analyzed using a factorial analysis with normalized varimax rotation. Two factors were above the eigenvalue threshold of one, and explained 56.8% of the variance (F1: 29.4%; F2: 27.4%). F1 combined U-CORT (weight = 0.7128) and B-CORT (weight = −0.7461), while F2 combined U-PGF (weight = −0.7020) and B-BDNF (weight = 0.6513).

The population was divided into two groups according to scores recorded for the three psychopathological assessments (the PCL, the HAD-D, and the HAD-A). The aim was to separate low-scoring (LS) soldiers who reported no psychopathological suffering (no scores below a cut-off) from high-scoring subjects (HS) with at least one score above a cut-off. Participants who scored above the cut-off on the HAD-D, the HAD-A, or the PCL were termed HAD-D^+^, HAD-A^+^, or PCLs^+^, respectively.

For the LS group, hierarchical tree clustering was applied, based on the four biological markers. Ward’s method was used for aggregation, and the Euclidean distance for distance calculation, after *z*-score normalization [58]. A four-cluster solution (C1, C2, C3, and C4) was selected as the best compromise between precision and discrimination in the context of four co-evolving variables, and comparisons were carried out between them. The C1 subgroup was considered as the Reference group, based on the normality of all considered variables. A factorial Analysis of Variance (ANOVA) was used for between-group comparisons followed, if necessary, by post hoc Bonferroni tests. Correlations were based on regression methods, and only results where *R^2^* > 0.10 were considered.

For the HS group, comparisons were carried out for each pathology, and with the LS group. The exception was PCLs+, which was also compared to the PCLs^+^ + HAD-A^+^ subgroup.

Results are expressed as mean ± standard error of the mean. Statistical significance was set at *p* < 0.05. Where the group was small, *t* < 0.10 was considered as evidence of a trend.

## 3. Results

### 3.1. Population Data

**Figure 1:**
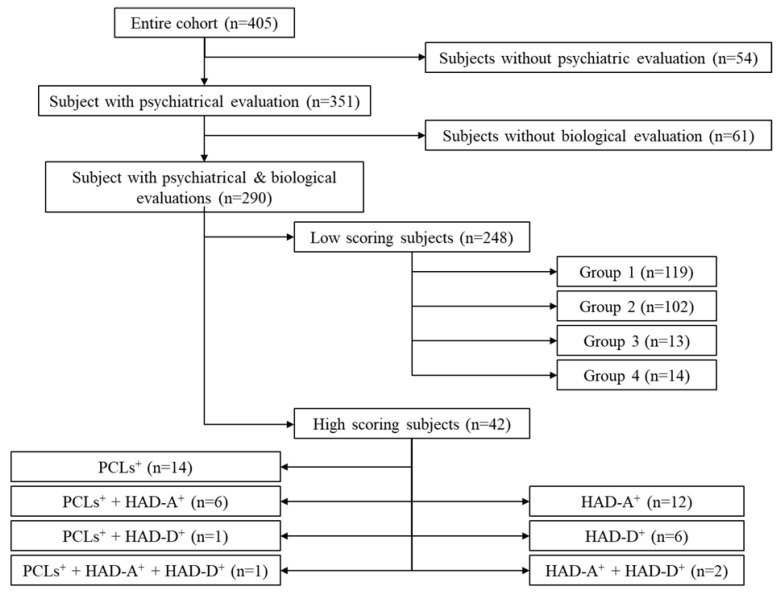
Flow diagram showing subpopulations within the initial cohort.

The studied cohort (Figure 1, *n* = 290) was extracted from the initial population (*n* = 405). Soldiers who did not fully complete the PCL, HADS-A and HADS-D questionnaires (*n* = 54) were excluded, as were those who did not provide full biological data (*n* = 61).

The flow chart shown in Figure 1 describes the two groups (LS and HS). The HS group consisted of 42 subjects who scored above the cut-off on the HAD-D, HAD-A, and/or PCL scales (Appendix A, Table A1). It should be noted that all of the soldiers in this group were able to cope with their everyday professional life, and had not received a clinical diagnosis. The LS group consisted of 248 soldiers characterized by scores below the cut-off on the PCL, the HAD-A, as the HAD-D (Appendix A, Table A2).

### 3.2. The HS Cohort

#### 3.2.1. Biological Characterization of the HS Subgroup

HS subjects were characterized by higher levels of U-PGF (*p <* 0.05) and U-CORT (*p =* 0.0763) than LS subjects (Table 1). 

Compared to LS subjects, HAD-D^+^ subjects had low B-BDNF concentrations (*p <* 0.05), while HAD-D^+^ + HAD-A^+^ subjects had higher B-BDNF (*p =* 0.0678) levels. Compared to LS subjects, PCLs^+^ subjects had higher U-CORT (*p <* 0.05) and U-PGF (*p <* 0.05) excretion, while PCLs^+^ + HAD-A^+^ subjects only had higher U-CORT excretion (*p =* 0.0507). The small size of the HS group meant that three subgroups: HAD-A^+^ + HAD-D^+^ (anxiety + depression); PCLs^+^ + HAD-D^+^ (PTSD + depression); and PCLs^+^ + HAD-A^+^ + HAD-D^+^ (PTSD+ anxiety + depression) were not considered for further analysis.

#### 3.2.2. Demographic Characterization of Pathological Subgroups

No difference in age, gender, family status, tobacco use, length of service, previous deployment, or number of deployments was observed between HS and LS soldiers.

Furthermore, no difference was observed for any pathology in comparison to LS subjects for any of these variables (Table 2).

#### 3.2.3. Psychological Characterization of HS Subgroups

In general, HS subjects scored higher than LS subjects on perceived stress (PSS, *p <* 0.001), alexithymia (TAS, *p <* 0.001), trait anxiety (STAI-T, *p <* 0.001), state anxiety (STAI-S, *p <* 0.001), burnout (BMS, *p <* 0.001), negative affect (PANAS-NA, *p <* 0.001), and general health (GHQ28, *p <* 0.001) scales (Table 3).

Compared to LS subjects, HAD-A^+^ subjects scored higher on PSS (*p <* 0.001), TAS (*p <* 0.01), STAI-T (*p <* 0.001), STAI-S (*p <* 0.001), BMS (*p <* 0.001), PANAS-NA (*p <* 0.001), PANAS-PA (*p <* 0.05), and GHQ28 (*p <* 0.001) scales (Table 4).

Compared to LS subjects, HAD-D^+^ subjects scored higher on PSS (*p <* 0.05), TAS (*p <* 0.01), STAI-T (*p =* 0.0522), and GHQ28 (*p <* 0.05) scales. However, PANAS-PA scores were lower (*p =* 0.05397).

Compared to LS subjects, PCLs^+^ subjects scored higher on PSS (*p* < 0.001), TAS (*p <* 0.01), STAI-T (*p <* 0.05), BMS (*p <* 0.001), PANAS-NA (*p <* 0.001), and GHQ28 (*p <* 0.001) scales. Similar results were observed for PCLs^+^ + HAD-A^+^ subjects (PSS: *p <* 0.001; TAS: *p <* 0.001; STAI-T: *p <* 0.001; STAI-S: *p <* 0.001; BMS: *p <* 0.001; PANAS-NA: *p <* 0.001; and GHQ28: *p <* 0.001).

Finally, compared to PCLs^+^ subjects, PCLs^+^ + HAD-A^+^ subjects scored higher on PCL (*p =* 0.06391), STAI-T (*p <* 0.05), STAI-S (*p <* 0.001), and PANAS-NA (*p <* 0.05) scales.

#### 3.2.4. Correlations

PCL scores did not correlate to any of the biological variables in the PCLs^+^ group.

### 3.3. The LS Cohort

#### 3.3.1. Biological Characterization of Clusters

The four clusters differed with respect to all four biological variables (Table 1). B-BDNF was only high in the C2 subgroup (*p <* 0.001 with C2 vs. C1: *p <* 0.001; C2 vs. C3: *p <* 0.01, and C2 vs. C4: *p <* 0.001). Similarly, U-CORT was only high in the C2 group (*p <* 0.001 with C2 vs. C1: *p <* 0.001; C2 vs. C3: *p <* 0.05, and C2 vs. C4: *p <* 0.001). U-PGF was only high in the C3 subgroup (*p <* 0.001 with C3 vs. C1: *p <* 0.001; C3 vs. C2: *p <* 0.001, and C3 vs. C4: *p <* 0.001). Finally, B-CORT was only high in the C4 subgroup (*p <* 0.001 with C4 vs. C1: *p <* 0.001; C4 vs. C2: *p <* 0.001, and C4 vs. C3: *p <* 0.001).

#### 3.3.2. Demographic Characterization of LS Subgroups

The C3 subgroup differed slightly from the C1 subgroup with respect to marital status (Table 2, *p =* 0.0535). The C4 subgroup differed significantly from the C1 subgroup: subjects were female (gender, *p <* 0.001), younger (age, *p <* 0.05), with a shorter length of service (*p <* 0.05) and less experience of deployment (previous deployment, *p <* 0.05).

#### 3.3.3. Psychological Characterization (Table 3 and Table 4)

Scores for LS subjects were very far from PCL, HAD-A and HAD-D thresholds. Scores for C2, C3, and C4 subgroups did not differ from the C1 subgroup for the PCL, HAD-A or HAD-D questionnaires (Table 2A).

Compared to C1 subjects, subjects in the C2 subgroup tended to score lower on the STAI-T (*p =* 0.06840), but higher on the GHQ28 (*p <* 0.05) scales. Subjects in the C3 subgroup scored lower than those in the C1 subgroup on the PSS scale (*p <* 0.05). Conversely, scores for subjects in the C4 subgroup were higher than for those in the C1 subgroup for the STAI-S (*p <* 0.05), and the PANAS-NA (*p <* 0.05) scales.

#### 3.3.4. Correlations

In order to understand the underlying biological mechanisms, we looked for inter-parameter correlations. This revealed that BDNF was slightly correlated to U-CORT in the C1 (*r*^2^ = 0.13, *p* < 0.001) and C2 (*r*^2^ = 0.11, *p* < 0.001) subgroups, but not C3 and C4 cohorts. With respect to the C2 subgroup, no correlation was observed between B-BDNF and GHQ28 as between U-CORT and GHQ28. In the C3 subgroup, no correlation was observed between the level of 8-iso-PGF2α and the PSS score. In the C4 subgroup, no correlation was observed between B-CORT levels and the STAI-S score.

## 4. Discussion

This study compared several types and levels of biological mediators of allostatic load with ecological data in a military cohort during pre-deployment. The main result is that around 10% of soldiers reported high scores on questionnaires that screen for anxiety, depression, and PTSD in the general population. The biological alterations associated with these profiles raise the question of how to detect pathologies in people who are considered to be well-adapted to their environment. Moreover, some of the soldiers who reported low scores on the same questionnaires also presented biological abnormalities. This latter finding raises the question of the value of detecting infra-clinical biological scars.

### 4.1. High Scoring Subjects

Ten percent of our apparently healthy cohort reported high scores on the HAD-A, HAD-D, and PCL questionnaires. This prevalence is lower than that observed in active service soldiers in the United States army [10,59]. The discrepancy can be explained by the poor sensitivity of the HAD questionnaire (for a cut-off of 11, sensitivity = 0.560 but specificity = 0.921 [37]), and the PCL (for a cut-off of 44, sensitivity = 0.944 and specificity = 0.844 [39]). Therefore, we probably underestimated the number of high-scoring subjects.

The fact that some soldiers reported high scores on these questionnaires, while being considered as healthy after a medical evaluation, deserves discussion. The medical evaluation not only assesses criteria with respect to certain pathologies [38], but also takes into account subjective complaints, the need for care, and the soldier’s adjustment to social and professional requirements.

#### 4.1.1. Sub-Depressive HAD-D^+^ Subjects

Sub-depressive subjects were those who scored high on the HAD-D, but remained well-adapted to the environment. They presented evidence of mental suffering (scores above the threshold of the GHQ28 [57]), with a lack of positive thoughts (scores below the threshold of the PANAS-PA [50]), and numbed emotional perception (scores lower than suffering subjects measured by the TAS [44]).

In this group, blood BDNF values were low, for all ages [60]. Low B-BNDF levels are observed in patients who are clearly suffering from depression [61,62,63,64,65,66], and healthy subjects suffering from neuroticism [67,68], especially those who have been recently, but not chronically exposed to stress [69]. Since stress exposure is known to contribute to depression [32,70,71], it is possible that members of this group are either recovering from recent exposure to stress, or are in the early phase of depression.

#### 4.1.2. Anxious HAD-A^+^ Subjects

Participants in the HAD-A^+^ subgroup exhibited high anxiety (higher scores on the PSS, STAI-T and STAI-S scales than healthy subjects) and mental suffering (higher scores on the GHQ28 scale than healthy subjects) with evidence of burnout (higher scores on the BMS scale than healthy subjects), more negative (higher scores on the PANAS-NA than healthy subjects) and fewer positive thoughts (lower scores on the PANAS-PA than healthy subjects) and alexithymia (higher scores on the TAS than healthy subjects). However, no specific biological signature was observed.

#### 4.1.3. Traumatized PCLs^+^ Subjects

PCLs^+^ subjects reported greater perceived stress (higher scores on the PSS than healthy subjects) and a background level of anxiety (higher scores on the STAI-T than healthy subjects). They report intense mental suffering (higher scores on the GHQ28 than healthy subjects), together with alexithymia (higher scores on the TAS than healthy subjects), burnout (higher scores on the BMS than healthy subjects) and more negative thoughts (higher scores on the PANAS-NA than healthy subjects).

This group is characterized by a chronic stress pattern with high nocturnal excretion of free radicals and cortisol. The few articles that have investigated free radical production in PTSD suggest that levels may be high [72,73,74], but not always [74]. In any case, an increase in free radical production tends to be related to exposure to intense stress, while it may be enhanced in patients suffering from PTSD [73,75], independent of their score on the PCL [72]. An increase in nocturnal urinary cortisol excretion is difficult to detect in PTSD subjects [76], and the archetypal description suggests low HPA activity [77,78]. Since high HPA activity is considered as protective [79], this suggests that members of the PCLs^+^ group have been traumatized, and are reacting to a risk of transition to PTSD.

Furthermore, the prevalence of this group (5% of our population) is similar to the 4% described in a population of students [80], and congruent with the detection of new-onset PTSD in a military population [81,82]. The epidemiology and biological signature suggest that members of this group have been exposed to intense stress, and are suffering from sub-chronic stress during a period of recovery. The fact that they are able to maintain normal social and professional relations without any medical intervention also supports this explanation, and may explain why a cut-off of 44 on the PCL overestimates the prevalence of PTSD [40].

The combination of anxiety and a high PCL score degrades the situation. Increased anxiety (STAI-T and STAI-S) and severe trauma (PCL) increase mental suffering, reflected in higher scores on the PANAS-NA. In our study, HAD-A^+^ subjects did not record high scores on the PCL (Table A1), but PCLs^+^ subjects did record high scores on the HAD-A. This finding suggests that while anxiety and traumatization are independent processes, they can be associated, consequently leading to a more severe pathology.

### 4.2. Low Scoring Subjects

#### 4.2.1. Subgroup Analysis

Participants who recorded low scores on the HAD-A, HAD-D, and PCL are considered the healthy reference. However, clustering based on biological data led to the determination of four subgroups (C1, C2, C3, and C4) with the C1 subgroup being assumed to be the reference for normal functioning. As subgroup comparisons were carried out between healthy subjects, it should be kept in mind that any statistical differences are often within the normal range. However, it is possible to isolate three physiological patterns in response to environmental challenges.

#### 4.2.2. The Three Patterns

The first pattern is characterized by a relationship between B-BNDF and glucocorticoid. It is observed in C1 and C2 subgroups, where the same correlation between the two variables was identified. The only difference between these two subgroups was the level of regulation; in particular, levels of B-BDNF and U-CORT were higher in the C2 group than the C1 group. This difference could be a marker of heterostasis, as the higher levels were in the range observed in anxious HAD-A^+^ and PCLs^+^ subjects, respectively. Furthermore, they are associated with psychological signs of suffering, as C2 subjects reported distress (high scores on the GHQ28), but not anxiety (low scores on the STAI-T).

The second pattern is observed in the C3 subgroup. This group reported little perceived stress (low scores on the PSS scale), together with increased urinary free radical excretion (U-PGF), with no evidence of mental distress. This can be interpreted as a form of physiological inhibition of stress perceptions, which is the consequence of free radical activity [83].

The third, and final pattern concerns the C4 subgroup. This group is very specific as it mainly consisted of young women with little experience of active duty or overseas deployment. Their high B-CORT concentration reflects a high level of stress and arousal, which is consistent with the context at the time when the blood samples were taken. Our finding is in accordance with the literature, which shows that females exhibit greater HPA reactivity [84] and arousal compared to males [85], and explains why females are more prone to stress-induced pathologies such as anxiety, panic, and insomnia than males [85]. Although we did not record whether these women were taking an oral contraceptive, this could be a confounding factor, as oral contraceptives tend to reduce the HPA response [86]. Psychological assessments confirmed a high level of anxiety, notably high scores on the STAI-S, but not the STAI-T. This intense stress response is neither related to increased perceived stress (scores on the PSS are similar to C1 subjects), nor poor mental health (scores on the GHQ28 are normal), despite a high level of negative thoughts. Taken together, this pattern suggests an anticipatory anxiety mechanism [87].

### 4.3. Study Limitations

Main limitations concern the sample. First, data from 115 included subjects (28.4%) could not be analyzed; 54 subjects (15.33) completed the questionnaires incorrectly, making their inclusion in the study protocol unusable; and 61 subjects (15.06%) did not have biological sampling. These observations reflect the difficulty of conducting a field study in the final operational preparations before deployment. Second, the lack of women in this study, which was due to the cohort’s characteristics, limits the extrapolation of our results to the general population. However, the hierarchical analysis did identify a cluster exclusively composed of women, which suggests that their number was sufficient to describe at least one pattern.

## 5. Conclusions

The analysis of our population of soldiers, who had been deemed medically fit for deployment, identified a graduated physiological response to environmental challenges: three patterns of physiological response were observed in low scoring subjects, while high scoring subjects reported moderate psychological suffering that did not impact their environmental and social adaptation. Although the analysis could be extended to psychiatric inpatients, this was beyond the scope of our study. We examined a large panel of outpatients, with more-or-less good adjustment to society, but who are paying a notable psychological price. We recommend that subthreshold clinical screening should be introduced, as it is able to link psychological suffering with biological markers. The correlation between allostasis and individual suffering in our professional group suggests that a model can be developed to build on the strengths of allostasis and traditional stress evaluation. The latter could be considered as an avenue for evidence-based prevention [88]. Our study opens the door to a new type of design for future prospective studies. The risk and indicators that drive stress-related pathologies must be considered as a function of the basal allostatic load, and this approach will make it possible to propose a tailored prevention program.

## Figures and Tables

**Table 1:** Factorial analysis of B-BDNF (ng/mL), U-PGF (pg/mL), U-CORT (μg/24 h) and B-CORT (mmol/mL). Groups refer to subjects who scored high on HAD-A (HAD-A^+^), HAD-D (HAD-D^+^) and PCL (PCLs^+^) scales. Comparisons between HS and LS groups used a factorial ANOVA and results are expressed as q: *p* < 0.10 and *#: p* < 0.05. Comparisons between pathologies and healthy subjects were carried out using a factorial ANOVA and results are expressed as *t* < 0.10 and *: *p* < 0.05. In the LS group, C2, C3 and C4 were compared to C1 using a factorial ANOVA, and results are expressed as: *** *p* < 0.001. Values are expressed as mean ± SEM.

Experimental Group	*n*	U-CORT	B-CORT	B-BDNF	U-PGF
Factorial analysis		Factor 1		Factor 2	
High scoring subjects (HS)	42	61.31 ± 10.76 q	605.24 ± 23.03	13.20 ± 1.11	22,683 ± 4375 #
HAD-D^+^	6	40.76 ± 8.24	579.83 ± 54.20	8.07 ± 2.33 *	23,796 ± 15,482
HAD-A^+^	12	50.90 ± 9.42	607.33 ± 45.87	15.41 ± 1.68	20,272 ± 6726
HAD-D^+^ + HAD-A^+^	2	37.81 ± 0.26	540.50 ± 101.50	20.61 ± 13.05 t	11,235 ± 645
PCLs^+^	14	74.21 ± 31.00 *	624.37 ± 47.66	11.44 ± 1.66	30,344 ± 8932 *
PCLs^+^ + HAD-A^+^	6	72.41 ± 11.90 t	605.50 ± 41.58	13.18 ± 1.63	17,193 ± 10,782
PCLs^+^ + HAD-D^+^	1	44.53	450	17.40	11,270
PCLs^+^ + HAD-D^+^ + HAD-A^+^	1	66.19	748	23.45	4944
Low scoring subjects (LS)	248	50.55 ± 1.71 q	597.74 ± 10.26	13.37 ± 0.35	15,212 ± 1353 #
C1	119	36.80 ± 1.32	578.85 ± 9.12	11.10 ± 0.32	10,523 ± 908
C2	102	68.49 ± 2.69 ***	556.25 ± 10.89	16.56 ± 0.61 ***	11,678 ± 931
C3	13	49.38 ± 8.85	576.08 ± 46.36	11.36 ± 0.97	96,018 ± 2797 ***
C4	14	37.89 ± 7.27	1080.64 ± 36.20***	11.25 ± 0.54	5787 ± 1506

**Table 2:** Demographic data as a function of experimental group. C2, C3, and C4 results for the LS group were compared to C1 using a factorial ANOVA for continuous variables, and a Chi^2^ test for non-continuous variables. In all cases, results are expressed as *t* < 0.10, with * *p* < 0.05; and *** *p* < 0.001. Values are expressed as mean ± SEM.

Experimental Group	Gender (M/F)	Age	Family (Yes/No)	Tobacco Use (Yes/No)	Length of Service	Previous Deployment (Yes/No)	Number of Deployments
High scoring subjects (HS)	38/4	29.9 ± 1.1	26/15	14/22	9.0 ± 1.1	29/12	3.9 ± 0.4
HAD-D^+^	6/0	26.5 ± 2.9	3/3	3/1	5.8 ± 2.0	4/2	3.5 ± 1.5
HAD-A^+^	10/2	30.7 ± 1.9	8/4	4/7	10.8 ± 1.9	10/2	3.3 ± 0.8
HAD-D^+^ + HAD-A^+^	2/0	22.0 ± 1.0	1/1	0/2	3.0 ± 2.0	1/1	4
PCLs^+^	12/2	28.4 ± 1.7	8/6	5/7	8.9 ± 1.6	10/4	4.9 ± 0.7
PCLs^+^ + HAD-A^+^	6/0	30.7 ± 4.7	5/1	1/4	11.7 ± 4.6	3/3	4.0 ± 1.4
PCLs^+^ + HAD-D^+^	1/0	33	0/1	1/0	13	1/0	2
PCLs^+^ + HAD-D^+^ + HAD-A^+^	1/0	-	-	0/1	-	-	-
Low scoring subjects (LS)	212/35	29.9 ± 0.4	133/113	89/133	9.3 ± 0.4	166/81	3.7 ± 0.2
C1	103/16	30.0 ± 0.6	58/61	50/53	9.3 ± 0.6	82/37	3.8 ± 0.3
C2	98/4	30.4 ± 0.7	61/41 t	30/62	9.8 ± 0.7	70/31	3.7 ± 0.3
C3	11/2	29.5 ± 1.9	10/3 t	8/4	9.3 ± 1.6	9/4	3.0 ± 0.4
C4	0/13 ***	25.6 ± 0.8 *	5/8	1/11	5.1 ± 0.7 *	5/9 *	1.6 ± 0.4

**Table 3:** Psychological evaluation of the stress response as a function of the experimental group. Results are expressed as t < 0.10, * *p* < 0.05; ** *p* < 0.01 and *** *p* < 0.001. Values are expressed as mean ± SEM.

Experimental Group	Size	PSS	TAS	STAI-T	STAI-S
High scoring subjects (HS)	42	39.9 ± 0.8 ***	43.3 ± 1.3 ***	43.3 ± 1.3 ***	40.1 ± 1.5 ***
HAD-D^+^	6	38.8 ± 4.0 *	31.2 ± 2.8 **	41.2 ± 4.6 t	37.4 ± 5.4
HAD-A^+^	12	39.7 ± 1.2 ***	44.2 ± 2.1 **	46.2 ± 2.4 ***	41.4 ± 2.7 ***
HAD-D^+^ + HAD-A^+^	2	38.5 ± 3.5	46.0 ± 1.0	46.5 ± 1.5 *	47.0 ± 3.0 **
PCLs^+^	14	39.6 ± 1.3 ***	44.2 ± 1.8 **	39.0 ± 2.2 *	35.7 ± 2.7
PCLs^+^ + HAD-A^+^	6	42.7 ± 2.4 ***	49.5 ± 3.8 ***	48.5 ± 2.8 ***	48.5 ± 2.5 ***
PCLs^+^ + HAD-D^+^	1	38	51	41	30
PCLs^+^ + HAD-D^+^ + HAD-A^+^	1	42	38	41	45
Low scoring subjects (LS)	248	32.9 ± 0.4	38.8 ± 0.4	34.5 ± 0.5	32.2 ± 0.5
C1	119	33.0 ± 0.6	38.9 ± 0.6	35.3 ± 0.7	31.6 ± 0.7
C2	102	33.0 ± 0.5	38.5 ± 0.7	33.4 ± 0.7 t	32.0 ± 0.6
C3	13	28.5 ± 2.0 *	38.9 ± 1.7	32.5 ± 2.0	30.7 ± 1.7
C4	14	34.8 ± 1.5	40.6 ± 1.6	36.9 ± 2.1	38.4 ± 3.1 *

**Table 4:** Scores on psychological health assessment questionnaires as a function of the experimental group. Results are expressed as t < 0.10, * *p* < 0.05; ** *p* < 0.01 and *** *p* < 0.001. Values are expressed as mean ± SEM.

Experimental Group	Size	BMS	PANAS-NA	PANAS-PA	GHQ28
High scoring subjects (HS)	42	27.6 ± 1.1 ***	25.0 ± 1.1 ***	37.1 ± 1.0	28.6 ± 1.9 ***
HAD-D^+^	6	22.7 ± 5.3	18.6 ± 2.1	33.63.8 t	24.0 ± 6.0 *
HAD-A^+^	12	26.6 ± 1.2 ***	26.7 ± 1.2 ***	34.9 ± 1.7 *	26.6 ± 2.8 ***
HAD-D^+^ + HAD-A^+^	2	25.5 ± 2.5	28.5 ± 5.5 **	32.0 ± 4.0	31.5 ± 0.5 **
PCLs^+^	14	28.4 ± 1.5 ***	22.4 ± 1.9 ***	38.2 ± 1.6	27.9 ± 3.9 ***
PCLs^+^ + HAD-A^+^	6	32.7 ± 3.5 ***	31.8 ± 3.4 ***	42.2 ± 2.2	36.5 ± 3.3 ***
PCLs^+^ + HAD-D^+^	1	33	19	41	28
PCLs^+^ + HAD-D^+^ + HAD-A^+^	1	-	32	41	37
Low scoring subjects (LS)	248	18.0 ± 0.5	17.7 ± 0.3	38.6 ± 0.4	15.4 ± 0.5
C1	119	18.6 ± 0.8	17.8 ± 0.4	38.4 ± 0.4	14.6 ± 0.7
C2	102	17.3 ± 0.8	17.1 ± 0.4	38.6 ± 0.5	16.8 ± 0.8 *
C3	13	15.0 ± 2.1	18.5 ± 1.4	40.8 ± 1.2	12.3 ± 2.8
C4	14	20.0 ± 1.8	20.8 ± 1.0 *	38.3 ± 1.1	15.3 ± 1.6

## Data Availability

The data presented in this study are available on request from the corresponding author. Data are not publicly available as they concern a military population.

## Appendix A

**Table A1:** Scores on pathological scales recorded by subjects who scored above thresholds. Values are given as mean ± SEM. HS: high scores.

Experimental HS Groups	*n*	PCLs	HAD-A	HAD-D
HAD-D^+^	6	26.8 ± 4.3	7.5 ± 0.7	11.8 ± 0.5
HAD-A^+^	12	30.9 ± 2.3	12.2 ± 0.4	6.0 ± 0.6
HAD-D^+^ + HAD-A^+^	2	27.0 ± 10.0	11.5 ± 0.5	11.0
PCLs^+^	14	50.2 ± 0.7	7.2 ± 0.7	5.2 ± 0.9
PCLs^+^ + HAD-A^+^	6	55.2 ± 3.4	13.5 ± 0.7	6.2 ± 1.4
PCLs^+^ + HAD-D^+^	1	53	8	11
PCLs^+^ + HAD-D^+^ + HAD-A^+^	1	50	18	13

**Table A2:** Scores on pathological scales recorded by subjects who scored below thresholds. Values are given as mean ± SEM. LS: low scores.

Experimental LS Groups	*n*	PCLs	HAD-A	HAD-D
All LS subjects	248	22.6 ± 0.4	5.2 ± 0.1	2.9 ± 0.1
C1	119	22.8 ± 0.7	5.2 ± 0.2	3.1 ± 0.2
C2	102	22.5 ± 0.7	5.1 ± 2.1	2.8 ± 0.2
C3	13	24.0 ± 2.1	5.5 ± 0.7	2.6 ± 0.5
C4	14	20.6 ± 1.6	5.5 ± 0.5	3.1 ± 0.5
